# Assessing malaria risk at night-time venues in a low-transmission setting: a time-location sampling study in Zambezi, Namibia

**DOI:** 10.1186/s12936-019-2807-x

**Published:** 2019-05-22

**Authors:** Jerry O. Jacobson, Jennifer L. Smith, Carmen Cueto, Mukosha Chisenga, Kathryn Roberts, Michelle Hsiang, Roly Gosling, Davis Mumbengegwi, Adam Bennett

**Affiliations:** 1 Malaria Elimination Initiative, Global Health Group, University of California, San Francisco, 550 16th St., San Francisco, CA 94158 USA; 2 Multidisciplinary Research Center, University of Namibia, Windhoek, Namibia; 3 Department of Pediatrics, Benioff Children’s Hospital, University of California San Francisco, San Francisco, CA USA; 4 Department of Pediatrics, University of Texas Southwestern Medical Center, Dallas, TX USA

**Keywords:** Malaria, Surveillance, Time-location sampling, High-risk populations, Namibia

## Abstract

**Background:**

Identifying efficient and effective strategies to reach and monitor populations at greatest risk of malaria in low-transmission settings is a key challenge for malaria elimination. In Namibia’s Zambezi Region, transmission is ongoing yet its drivers remain poorly understood. A growing literature suggests that night-time social activities may lead to malaria exposure that is beyond the reach of conventional preventive interventions, such as insecticide treated bed nets and indoor residual spraying.

**Methods:**

Formative research was conducted with community members in March, 2015 in the catchment areas of six randomly selected health facilities in the western Zambezi Region to identify night-time locations where large numbers of individuals regularly congregate. Using time-location sampling, a survey was conducted between March and May, 2015 at community-identified venues (bars and evening church services) to develop representative estimates of the prevalence of parasite infection and risk factors among venue-goers.

**Results:**

When compared to a contemporaneous household survey of the general population aged 15 and older (N = 1160), venue-goers (N = 480) were more likely to have spent the night away from their home recently (17.3% vs. 8.9%, P = 0.008), report recent fever (65.2% vs. 36.9%, P < 0.001), and were less likely to have sought care for fever (37.9% vs. 52.1%, P = 0.011). Venue-goers had higher, but not significantly different, rates of malaria infection (4.7% vs. 2.8%, P = 0.740). Risk factors for malaria infection among venue-goers could not be determined due to the small number of infections identified, however self-reported fever was positively associated with outdoor livelihood activities (adjusted odds ratio [AOR] = 1.9, 95% CI 1.0–3.3), not wearing protective measures at the time of the survey (AOR = 6.8, 9% CI 1.4–33.6) and having been bothered by mosquitos at the venue (AOR = 2.7, 95% CI 1.5–4).

**Conclusions:**

Prevention measures and continued surveillance at night-time venues may be a useful complement to existing malaria elimination efforts.

## Background

Namibia is a country with low malaria transmission that has successfully reduced the burden of malaria over the past 15 years through the large-scale deployment of long-lasting insecticide-treated nets (LLINs), indoor residual spraying (IRS), artemisinin-based combination therapy (ACT), and improved availability of rapid diagnostic tests (RDTs) [1, 2]. Malaria cases and deaths decreased by 98 percent over this period, far surpassing targets and enabling the implementation of elimination strategies.

Although Namibia aims to eliminate malaria by 2022, recent successes have plateaued, with outbreaks commonplace over the past 2 years. Transmission is known to be clustered in the northern regions of the country [1, 3], but evidence of the specific drivers of remaining transmission and how to address them would help guide elimination efforts. Lower transmission settings like northern Namibia present unique challenges for surveillance given the small number of infected individuals and increased heterogeneity of transmission both spatially and socially. In some malaria elimination settings, outdoor social and occupational behaviours are thought to lead to increased risk by increasing vector contact outside the home [3–5], thus reducing the effectiveness of household-based preventive interventions, such as IRS and LLINs.

Identifying the circumstances and behaviours that lead to increased risk of infection, as well as how to effectively reach subgroups at increased risk, is essential to reduce malaria transmission. Using time-location sampling (TLS), this study investigated whether locations where individuals tend to gather away from their homes during mosquito-biting hours could prove useful sites to conduct active surveillance and prevention activities in the Zambezi Region. TLS is a multi-stage cluster sampling method in which individuals are recruited from randomly selected venues during randomly selected venue-day-time intervals (VDTs), in order to develop representative estimates of the prevalence of malaria, risk behaviours and prevention uptake among venue-goers [6, 7]. TLS is frequently used in surveillance of populations at elevated risk for HIV [8]. A similar form of venue-based sampling (without randomization of times) identified excess malaria risk among seasonal migrant agricultural workers in Ethiopia [9].

## Methods

### Study site

This study was conducted in six randomly selected health facility catchment areas (HFCAs), out of 13 total HFCAs in the western Zambezi Region in northeastern Namibia. The Zambezi Region is arid and sparsely populated and borders Zambia and Angola to the north and Botswana to the south. Malaria transmission is seasonal, peaks between December and April and is highly dependent on rainfall patterns. This study was conducted in the western Zambezi Region, where prevalence is highest and access to services lowest. The population was 35,381 based on 2011 census data [10] and the annual parasite index was 26 per 1000 persons in 2014, according to health information system data and a census conducted by the project partners.

The study was conducted simultaneously with a representative household survey in the same catchment areas [11], a facility-based case–control study (Smith et al. [12], pers.commun.), and a peer-referral study to examine clustering of malaria risk within social networks.

### Formative assessment

A formative assessment was conducted in March, 2015 to identify types of venues that were active between dusk and dawn, to identify their peak attendance times, and to engage with the community and venue owners regarding the potential acceptability and feasibility of the venue-based study design.

In each HFCA, one focus group discussion (FGD) was held with community leaders (“headmen”). Additionally, one FGD was held with community health volunteers in the combined study area. All FGDs were conducted in the Siliozi language and followed a semi-structured guide. Participants were asked to discuss the types of venues that would be likely to meet a set of pre-defined venue eligibility criteria, to identify potential challenges to recruitment, and to develop a map of such venues and a list of peak attendance times. The venue eligibility criteria included that the venues be open at least once weekly between sundown and sunrise, have clientele mostly older than age 14 years, have a peak attendance of at least 10 people, have clientele deemed likely to be willing to participate, and that carrying out the study would not be expected to pose a risk to survey staff. Based on findings from the FGDs, a limited number of types of venues that appeared to have the largest number of venue-goers, greatest duration of time outdoors during mosquito-biting hours, and accessibility, were selected for inclusion in the survey. Formative study staff then visited the venues identified to confirm peak attendance times, request permission to conduct the study, and identify locations within or outside of each venue to conduct malaria testing and confidential interviews.

### Sampling and recruitment

TLS was used to select venues and times to recruit survey participants. A sampling frame of VDTs was first constructed. VDTs were defined as 2- to 4-h intervals of peak attendance at a specific venue (e.g., Bar #1 from 7 to 9 pm on Fridays). At the start of each month, VDTs were randomly selected from the sampling frame and scheduled onto a calendar of recruitment events. As possible, four events (two per each of two field teams) were scheduled each Wednesday, Friday and Saturday (days when peak venue attendance was identified) during the approximately 10-week data collection period. VDTs were selected by simple random sampling subject to logistical constraints: to limit travel time, a field team could travel to only one HFCA per evening; to limit burden on venue-goers, a venue could be visited no more than once weekly. An alternate VDT beginning on or after the start of each of these primary VDTs was also randomly selected, in case the primary venue was found to be closed, inaccessible, or had low attendance. Sampling quota were a minimum of 11 and a maximum of 25 participants per event, to meet a planned total sample size of 800 participants. The sample size was chosen to provide 80% power to detect a difference in polymerase chain reaction (PCR) parasite prevalence of 4 percentage points between households (anticipated to be 2%) and venues (anticipated to be 6%), assuming a design effect of 2 due to clustering by sampling event. Each month, the sampling frame was updated to reflect new venues or venue closures.

Field teams included an enumerator, a nurse and two interviewers. Enumerators designated an intercept line to count venue-goers present at any time during the event. When not conducting interviews, interviewers approached all venue-goers who crossed the line and verbally assessed eligibility.

Males and females ages 15 years and older who lived or worked in the Zambezi Region, had not participated previously and did not appear too intoxicated to provide informed consent were assessed for eligibility using a screening form and, if deemed eligible, invited to a confidential area outside and nearby the venue entrance. Those willing to provide written informed consent then completed a 15- to 30-minute interviewer-administered questionnaire via electronic tablet, which included demographics, outdoor activities between dusk and dawn, protective measures against mosquitos at the venue and place of sleeping, fever and malaria history, treatment-seeking for fever, and venue attendance patterns. The nurse then collected a finger-prick blood sample, conducted a malaria RDT and prepared a dried blood spot for molecular testing.

Participants with positive RDT results were provided free treatment on-site. Blood samples were frozen before being sent to a laboratory in Windhoek for loop-mediated isothermal amplification (LAMP), and follow-up PCR following all positive LAMP results, and on 10% of negatives. No monetary or other incentives were provided.

Participant ID codes linked questionnaires, specimens and test results. Names were not recorded. The study protocol was approved by The University of California, San Francisco Committee on Human Research, Institutional Review Board, the University of Namibia Research Ethics Committee, and the Research Management Committee of the Ministry of Health and Social Services of Namibia.

### Household survey

Estimates of characteristics of the population reached by TLS were compared to results from a cross-sectional household survey covering the same geographic region and conducted between April and June, 2015. In this survey, 684 households were randomly sampled, 529 households enrolled, and 1918 household members interviewed and tested for malaria [11]. Sampling in the household survey was with probability proportional to number of households across HFCAs.

### Measures

Responses to the following question were grouped to differentiate indoor and outdoor livelihood activities: “*What are the occupations or activities that you do to support yourself and/or your family with income or food*?”. Outdoor activities were defined as agriculture or fishing (from responses subsistence agricultural worker, agricultural labourer, skilled agricultural or fishery worker, subsistence fishery worker, and small-scale fish vendor) and construction or security guard. Indoor activities were defined as professional, student or merchant (from responses teacher, student, professional, retail merchant, and other sales and services), domestic work (“homemaker”) and “other” responses.

Respondents were asked about protective measures against malaria in their home including IRS and LLIN ownership, as well as whether they had been ill with fever anytime in the past 3 months, the last 2 weeks, and whether they sought treatment.

### Statistical analysis

For categorical measures, proportions and 95% confidence intervals (CIs) were estimated; medians and interquartile ranges were estimated for continuous measures. Differences by venue type were assessed using Wald tests. Differences between the TLS and household surveys were assessed using two-sample Z-tests.

Risk factors for malaria infection by RDT, PCR or LAMP could not be identified due to the small number of positives detected. Associations were identified between self-reported fever in the past 3 months using bivariate and multivariable logistic regression, examining variables related to demographics, potential exposure at the venue and at the place of sleeping. Response categories were combined in some cases where bivariate associations were similar. The multivariable model included all variables with bivariate associations at the 10% level. Analysis was conducted in Stata version 12.0 using procedures for complex surveys.

Estimates of prevalence and tests for associations from the TLS sample were weighted and standard errors were linearized to account for clustering by recruitment event [13–15]. Sampling weights were calculated as $\frac{1}{{F}_{e}{A}_{i}}$, where *F*_*e*_ was the sampling fraction from respondent (i)’s sampling event and *A*_*i*_ was the frequency of attendance, defined as the number of days the respondent went to the same type of venue (i.e., bars or churches) in the past 30 days between sundown and sunrise [16]. Frequency of attendance was imputed at the sample mean, by catchment area and venue type for 14 (2.9%) participants due to non-response. A 5% trim was applied to the weights due to outliers ( > 3 standard deviations from the mean). Differences at the 5% level were considered statistically significant.

## Results

### Identifying potentially high-risk venues

**Fig. 1:**
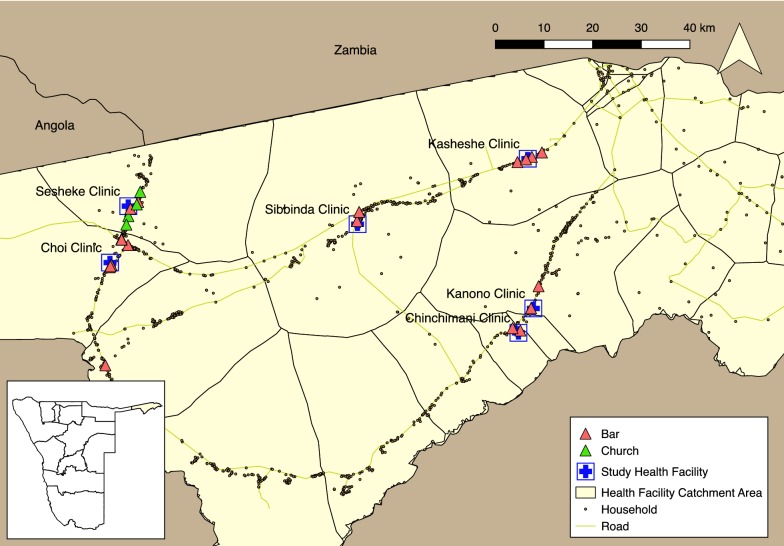
Study area, Zambezi Region, Namibia, 2015

Several types of locations where individuals gathered between sundown and sunrise were identified during FGDs, including bars, churches, construction work sites, and cattle, fishing and police camps. The TLS study was limited to bars and churches as they were considered the most well-attended and accessible locations, often have windows or doors that are left open, and in some cases incomplete walls or open eaves. Twenty-one bars and four churches with evening hours were identified within the 6 catchment areas. VDTs, defined based on reported peak times, were generally between 3 and 8 pm at churches and 6 pm and 12am at bars. Selected HFCAs, venues, and households in the study area are shown in Fig. 1.

### TLS recruitment

From March 27 to May 30, 2015, 480 study participants were recruited from 67 events held at 43 randomly selected VDTs, including 390 (81%) participants at bars and 90 (19%) at churches. Between 1 to 5 (median 3) sampling events were held per venue. Each venue contributed from 2 (0.4%) to 45 (9.4%) participants. Six additional events during the final month of data collection produced no new participants despite venue-goers in attendance. Of venue-goers approached, 80% at churches and 67% at bars were eligible and agreed to participate; this declined from 76% overall during the first 30 days to 51% during the latter half of the study period. The main reasons cited by field teams for non-participation were previous participation and lack of incentives.

### Demographics

Based on weighted analysis, an estimated 62.8% of individuals frequenting bars and evening church services were male and 37.2% were female (Table 1). The median age was 25 years and 26.1% were 35 years or older. Most had completed primary education (79.0%). Most venue-goers were Namibian citizens (97.9%), while 1.4% were Zambian. About one quarter (27.5%) worked in agriculture or fishing and 1 in 10 (11.0%) in construction or as security guards; 37% earned income from any of these activities. Bar patrons were more likely to be younger (median 25 vs. 36 years; *P* = 0.001), male (66.1% vs. 36.1%; *P* = 0.0281), and less likely to work in agriculture or fishing (17.8% vs. 52.9%; *P* = 0.014) than church-goers.

**Table 1:** Estimated characteristics of venue-goers, TLS survey, Zambezi Region, Namibia, 2015

Variables	All venue-goers (N = 480)			Bars (N = 390)			Churches (N = 90)		
	N	n	Percent (95% CI)	N	n	Percent (95% CI)	N	n	Percent (95% CI)
Demographics									
Sex									
Female	480	155	37.2 (28.0–47.3)	390	105	33.9 (24.0–45.5)	90	50	63.9 (49.2–76.5)
Male	480	325	62.8 (52.7–72.0)	390	285	66.1 (54.5–76.0)	90	40	36.1 (23.5–50.8)
Age									
Median (IQR)	480	-	25 (20–36)	390	-	25 (20–34)	90	-	36 (23–50)
15–24	480	202	46.8 (37.1–56.7)	390	176	49.1 (38.7–59.5)	90	26	27.7 (13.7–48.1)
25–34	480	133	27.1 (21.2–34.0)	390	116	28.3 (21.8–35.8)	90	17	17.7 (9.8–29.7)
35–44	480	77	15.2 (10.5–21.5)	390	61	14.1 (9.3–20.9)	90	16	24.1 (14.6–37.3)
45+	480	68	10.9 (6.6–17.4)	390	37	8.5 (4.6–15.4)	90	31	30.4 (17.9–46.7)
Education									
Less than primary	476	104	21.0 (15.6–27.7)	386	75	19.7 (14.1–26.7)	90	29	32.0 (20.6–46.1)
Primary	476	212	43.7 (35.6–52.2)	386	179	43.8 (35.0–53.0)	90	33	42.8 (24.4–63.4)
Secondary or above	476	160	35.3 (28.0–43.2)	386	132	36.5 (28.4–45.3)	90	28	25.2 (16.5–36.3)
Citizenship									
Namibiana^a^	480	462	97.9 (95.7–99.0)	390	376	98.5 (96.9–99.3)	90	86	93.2 (72.7–98.6)
Zambian	480	15	1.4 (0.7–3.0)	390	13	1.3 (0.6–3.0)	90	2	2.2 (0.3–16.1)
Other	480	3	0.7 (0.1–3.3)	390	1	0.2 (0.0–1.4)	90	2	4.6 (0.6–28.7)
Livelihood activities									
Agriculture/fishing	480	174	27.5 (20.0–36.5)	390	123	23.3 (16.4–31.9)	90	51	62.5 (42.7–78.9)
Construction/security guard	480	60	11.0 (7.5–15.9)	390	54	11.8 (7.9–17.3)	90	6	4.3 (1.3–13.0)
Professional/student/merchant	480	189	40.5 (33.1–48.4)	390	157	41.7 (33.9–49.9)	90	32	31.3 (15.2–53.7)
Homemaker/other	480	97	15.7 (10.8–22.3)	390	85	16.3 (10.9–23.7)	90	12	10.5 (3.8–25.6)
Agriculture, fishing, construction or security guard	480	219	37.0 (28.5–46.3)	390	164	33.7 (25.5–43.1)	90	55	64.1 (44.1–80.2)
Venue									
Attends this type of venue (bar or church) between sundown and sunrise at least weekly	466	294	28.8 (21.2–37.8)	383	245	28.5 (20.2–38.4)	83	49	31.4 (19.1–47.2)
Duration of typical visit to venue									
< 1 h	469	73	20.7 (14.7–28.3)	383	60	20.4 (14.1–28.6)	86	13	23.1 (9.3–46.7)
1–2 h	469	155	34.8 (25.6–45.4)	383	125	33.9 (24.1–45.5)	86	30	42.1 (22.5–64.7)
≥ 3 h	469	241	44.5 (35.0–54.3)	383	198	45.7 (35.5–56.2)	86	43	34.7 (19.8–53.5)
Ever bothered by mosquitos at venue	478	281	53.8 (44.7–62.7)	389	229	53.3 (43.2–63.1)	89	52	57.8 (46.3–68.5)
Personal protective measures against malaria at time of survey	478	10	2.5 (0.9–6.3)	388	9	2.7 (1.0–7.1)	90	1	0.5 (0.1–3.5)
Place of sleeping									
Last time IRS in structure where sleeps									
< 1 year	361	173	50.8 (43.0–58.6)	284	124	48.9 (40.5–57.2)	77	49	63.7 (48.2–76.8)
≥ 1 year	361	83	19.8 (14.5–26.4)	284	70	20.3 (14.5–27.7)	77	13	16.5 (8.2–30.5)
Never	361	105	29.4 (22.0–38.0)	284	90	30.8 (22.9–40.0)	77	15	19.8 (6.9–45.0)
Owns a mosquito net	480	204	43.7 (36.1–51.6)	390	166	44.6 (36.2–53.3)	90	38	35.8 (27.1–45.6)
Slept under a net last night	479	135	26.9 (20.3–34.8)	389	106	27.1 (19.8–36.0)	90	29	25.2 (18.8–33.0)
Slept outdoors in past 2 weeks	479	50	10.5 (7.1–15.3)	389	38	10.6 (6.9–15.9)	90	12	10.2 (5.0–19.8)
Spent the night outside of home village in past 4 weeks	479	82	17.3 (12.6–23.3)	389	65	17.0 (11.9–23.7)	90	17	20.0 (12.2–30.9)
Fever									
Past 2 weeks	478	245	52.1 (45.8–58.3)	388	191	50.4 (43.9–56.9)	90	54	65.7 (49.6–78.9)
Past 3 months	478	300	65.2 (59.2–70.8)	388	238	64.2 (57.7–70.2)	90	62	73.4 (59.2–83.9)
Sought treatment last time^b^	299	105	37.9 (28.9–47.8)	237	79	36.8 (27.0–47.8)	62	26	45.7 (28.8–63.6)
Treatment received last time^c^									
Coartem	94	5	3.4 (3.4–3.4)	68	4	3.8 (3.8–3.8)	26	1	1.3 (0.2–9.6)
SP/Fansidar	94	1	0.3 (0.3–0.3)	68	0	0.0	26	1	1.5 (0.2–10.2)
Panadol	94	61	69.2 (69.2–69.2)	68	44	68.5 (68.5–68.5)	26	17	72.1 (51.0–86.5)
Other	94	26	26.6 (26.6–26.6)	68	19	26.9 (26.9–26.9)	26	7	25.1 (10.8–47.9)
None	94	1	0.7 (0.7–0.7)	68	1	0.8 (0.8–0.8)	26	0	-
Malaria infection									
RDT reactive	479	3	0.5 (0.1–3.6)	389	1	0.0 (0.0–0.2)	90	2	4.6 (0.6–28.7)
PCR reactive	479	7	1.6 (0.5–4.7)	389	6	1.6 (0.5–5.2)	90	1	1.4 (0.2–11.1)
LAMP reactive	480	17	4.7 (2.4–8.9)	390	13	4.3 (2.0–9.0)	90	4	7.8 (2.1–25.1)

*CI* Confidence interval, *IRS* indoor residual spraying, *RDT* rapid diagnostic test, *PCR* polymerase chain reaction, *LAMP* loop-mediated isothermal amplification

^a^Includes 1 participant with Namibian and Zambian citizenship

^b^Among those with fever in past 3 months

^c^Among those who sought advice or treatment at last fever

### Exposure at venues

Nearly a third (28.8%) of participants frequented bars or churches between sundown and sunrise at least weekly. Most (79.3%) spent more than 1 h during a typical visit to the venue where they were surveyed and 44.5% spent 3 h or more. About half (53.8%) had been bothered by mosquitoes at the venue previously; 2.5% were using mosquito repellent at the time of the interview. These estimates of potential exposure to mosquito bites were similar at bars and churches.

### Exposure at place of sleeping

About half (50.8%) of venue-goers slept in structures the previous night where IRS had been conducted in the past year, 43.7% owned a mosquito net and 26.9% had slept under a net the previous night. Fifty participants (10.5%) had slept outdoors in the past 2 weeks and 82 (17.3%) had spent the night outside of their home village in the past 4 weeks. Of the 82 participants reporting overnight travel, 78 travelled within Namibia, 2 to Zambia and 2 did not report their destination. These estimates were also similar at bars and churches.

### Malaria infection, fever and treatment-seeking

The estimated prevalence of malaria infection was 4.7% by LAMP and 0.5% by RDT, with no differences by type of venue. Of the 17 LAMP-positive participants, 13 were recruited from bars (11 males and 2 females) and 4 from churches (3 males and 1 female); LAMP-positive participants’ ages ranged from 17 to 42 years (median 24 years) and were similar by sex and venue type. Only 2 of the 17 LAMP-positive participants tested positive by RDT. Two of the three RDT-positive participants tested positive by LAMP.

An estimated 52.1% of venue-goers had self-reported fever in the 2 weeks prior to the survey and 64.2% in the prior 3 months. Of the latter, 37.9% (105) sought treatment or advice for the most recent fever episode. None of those who reported fever in the past 3 months reported receiving chloroquine or quinine: five received Coartem®; one received SP/Fansidar®; 61 received Panadol®; and 26 received other unspecified treatments. Of the six participants with fever in the past 3 months who took Coartem or SP/Fansidar anti-malarials at last fever, none tested positive for malaria by RDT, PCR or LAMP. Of the 88 who received other or no treatments, five (5.7%) tested positive for malaria: two by RDT, one by PCR and five by LAMP; all five reported fever in the past 2 weeks.

Self-reported fever in the past 3 months was associated with engaging in livelihood activities that included agriculture, fishing, construction and security work (OR = 1.7; *P* = 0.030), ever being bothered by mosquitos at the venue (OR = 2.8; *P* < 0.001), not wearing personal protective measures when interviewed (OR = 6.5; *P* = 0.043), and Zambian citizenship (OR = 12.4; *P* = 0.009) (Table 2). Malaria infection by LAMP was not significantly associated with self-reported fever in the past 2 weeks or 3 months (P ≥ 0.4).

**Table 2:** Factors associated with fever in past 3 months, TLS survey, Zambezi Region, Namibia

Variables	Fever in past 3 months		Bivariate	Multivariable (N = 474)
	n	Percent	OR (95% CI)	AOR (95% CI)
Demographics				
Sex				
Female	100	68.0	1.0	
Male	200	63.6	0.8 (0.4–1.6)	
Age				
15–24	125	69.6	1.0	
25–34	88	66.6	0.9 (0.5–1.6)	
35–44	45	51.9	0.5 (0.2–1.0)	
45+	42	61.5	0.7 (0.3–1.7)	
Education				
Primary or above	229	63.4	1.0	
Less than primary	71	72.1	1.5 (0.6–3.5)	
Citizenship				
Namibian	284	64.6	1.0	1.0
Zambian	16	95.8	12.4 (2.1–71.2)	*12.2 (1.6–91.7)*
Livelihood activities include agriculture, fishing, construction, or security guard				
No	152	60.0	1.0	1.0
Yes	148	74.3	1.7 (0.9–3.3)	*1.9 (1.0–3.3)*
Venue				
Attends this type of venue (bar o church) between sundown and sunrise at least weekly				
No	107	63.8	1.0	
Yes	184	68.6	1.2 (0.8–2.0)	
Duration of typical visit to venue				
< 1 h	43	66.7	1.0	
1–2 h	108	74.8	1.5 (0.6–3.7)	
≥ 3 h	145	58.7	0.7 (0.3–1.5)	
Ever bothered by mosquitos at venue				
No	97	52.7	1.0	1.0
Yes	202	75.9	2.8 (1.7–4.8)	*2.7 (1.5–4.6)*
Wearing mosquito repellent, cream or spray at time of survey				
Yes	5	23.2	1.0	1.0
No	295	66.4	6.5 (1.1–40.4)	*6.8 (1.4–33.6)*
Place of sleeping				
Last time IRS in structure where sleeps				
< 1 year	109	64.3	1.0	
≥ 1 year	56	65.0	1.0 (0.5–2.1)	
Never	71	66.7	1.1 (0.5–2.3)	
Slept outdoors in past 2 weeks				
No	264	65.1	1.0	
Yes	35	65.9	1.0 (0.3–3.6)	
Owns a mosquito net				
No	180	68.9	1.0	
Yes	120	60.4	0.7 (0.4–1.3)	
Slept under a net last night				
No	222	64.9	1.0	
Yes	78	66.3	1.1 (0.5–2.4)	
Spent the night outside of home village in past 4 weeks				
No	247	63.9	1.0	
Yes	52	71.6	1.4 (0.6–3.3)	

*OR* Odds ratio, *AOR* unadjusted odds ratio, *CI* confidence interval, *IRS* indoor residual spraying

AORs significant at the 5% level are in italic

### Venue-goers versus household population

**Fig. 2:**
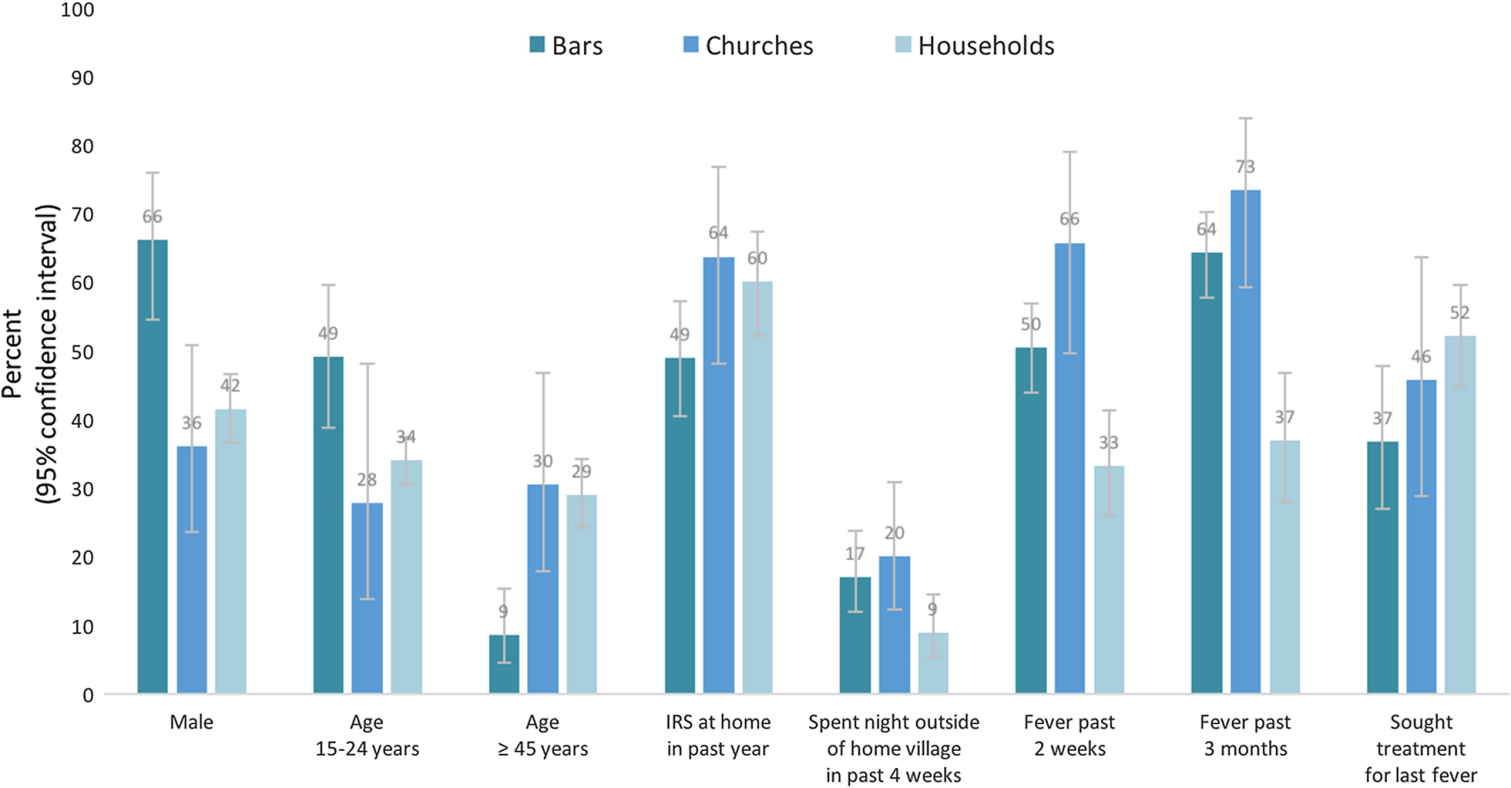
Variables with significant differences detected between TLS and household surveys, Zambezi Region, Namibia, 2015. Sample sizes were 390 (Bars), 90 (Churches), and 1160 (Households) with some non-response for specific variables (see Tables 1 and 3)

Relative to the household population aged 15 years and older, venue-goers were more likely to be male (62.8% vs. 41.5%; *P* < 0.001), aged 15 to 24 years (46.8% vs. 34.0%; *P* = 0.013), and less likely to be age 45 years and older (10.9% vs. 29.0%; *P* < 0.001) (Table 3). Venue-goers were more likely to have spent the night away from their home village in the past month (17.3% vs. 8.9%; *P* = 0.008), to have experienced fever in the past 2 weeks (52.1% vs. 33.2%; *P* < 0.001) and 3 months (65.2% vs. 36.9%; *P* < 0.001), less likely to have sought treatment at last fever (37.9% vs. 52.1%; *P* = 0.011), and moderately less likely to reside in a structure where IRS had been conducted in the past year (50.8% vs. 60.0%; *P* = 0.063). Prevalence of malaria infection by LAMP between the venue-going population and the general population was not significantly different (4.7% vs. 2.8%; *P* = 0.740). Bar and church sub-samples both differed from the general population with respect to prevalence of fever and spending the night away from home; bar-goers differed from the general population with respect to age (*P* < 0.001), sex (*P* < 0.001), IRS in past year (*P* = 0.031), and treatment-seeking for fever (*P* = 0.011) (Fig. 2).

**Table 3:** Comparison of estimates from TLS and household surveys, Zambezi Region, Namibia, 2015

Variables	TLS survey (N = 480)			Household survey (N = 1160)			P-value
	N	Percent	SE	N	Percent	SE	
Demographics							
Sex							
Female	480	37.2	0.049	1160	58.5	0.019	* < 0.001*
Male	480	62.8	0.049	1160	41.5	0.019	* < 0.001*
Age							
15–24	480	46.8	0.050	1160	34.0	0.014	*0.013*
25–34	480	27.1	0.032	1160	21.9	0.015	0.140
35–44	480	15.2	0.027	1160	15.2	0.008	1.00
45+	480	10.9	0.027	1160	29.0	0.019	* < 0.001*
Place of sleeping							
IRS in structure where sleeps in < 1 year	361	50.8	0.039	505^a^	60.0	0.030	0.063
Slept outdoors in past 2 weeks	479	10.5	0.020	1158	10.7	0.034	0.962
Owns a mosquito net	480	43.7	0.039	NA	NA	NA	NA
Slept under net last night	479	26.9	0.036	1154	34.5	0.036	0.140
Spent the night outside of home village in past 4 weeks	479	17.3	0.027	1160	8.9	0.017	*0.008*
Fever							
Past 2 weeks	478	52.1	0.032	1151	33.2	0.030	* < 0.001*
Past 3 months	478	65.2	0.029	1153	36.9	0.037	* < 0.001*
Sought treatment last time^b^	299	37.9	0.048	422	52.1	0.029	*0.011*
Received treatment last time^b^	288	33.9	0.047	422	38.6	0.492	0.924
Malaria infection (LAMP)	480	4.7	0.015	1064	2.8	0.055	0.740

SE, standard error; LAMP, loop-mediated isothermal amplification. P-value for differences significant at the 5% are in italic

^a^Variable reported by household heads only (N = 505)

^b^Among those with fever in past 3 months

## Discussion

In a low-transmission setting on the path to malaria elimination, community members identified types of locations where individuals gather away from their homes during late afternoon and evening mosquito-biting hours. Active surveillance was conducted at what appeared to be the most numerous and accessible venues for malaria surveillance—bars and churches with late afternoon and evening services—during randomly selected peak-attendance days and times. TLS proved a feasible and acceptable strategy to determine the prevalence of malaria infection, activities that may increase exposure, and use of personal protective measures among patrons of these venues.

This study was motivated by the hypothesis that partially outdoor locations where individuals gather during mosquito-biting hours may contribute to ongoing transmission in the western Zambezi region, yet findings on this point were mixed. While malaria prevalence among venue-goers was nearly twice that among the general population aged 15 years and older as assessed by a household survey, the difference was not statistically significant. Because the study did not reach its planned sample size, the lack of significance may be due to limited statistical power. If this is the case and venue patrons are truly at greater risk, then this study offers a number of explanations.

First, there is some evidence venue attendance itself may be a source of risk. Malaria rates were non-significantly higher at both bars and churches, compared to the general population, suggesting a common pattern. Most venue-goers had been bothered by mosquitoes while attending both types of venues, while few used protective measures. Both of these characteristics were associated with self-reported fever. Rates of fever were significantly greater compared to the general population and venue-goers were less likely to seek advice or treatment when experiencing fever, suggesting they could potentially transmit to peers over a longer period, including other venue-goers, compared to non-venue-goers. Recent evidence suggests alcohol consumption may attract anophelene mosquitoes [17], so that bar patrons may be at greater risk of potentially infectious bites. Yet, much of this evidence is tenuous: the study did not verify self-reported fever nor determine how participants assessed fever, so that this measure could merely reflect “feeling hot” or be due to other unspecified viral or bacterial causes, particularly given the low level of malaria transmission during the study period. Because entomological data were not collected around venues, the transmission potential of mosquitoes present could not be assessed.

A second explanation, more strongly supported by our data, is that the venues attract individuals who were otherwise at greater risk. Bar patrons were more likely to be male and aged 15–24, while patrons of both types of venues were more likely to engage in overnight travel outside of their community than the general population, characteristics which have been identified as risk factors for malaria elsewhere in northern Namibia [15]. Many venue-goers, principally evening church patrons, also engage in livelihood activities that are primarily outdoors and therefore may increase exposure.

A third, related explanation is that evening venue attendance may reflect a tendency to be outdoors at night generally. Venue attendance, together with the low observed levels of protective measures, may also reflect less concern about mosquito bite exposure, however such attitudes were not measured directly. Together, study findings point to a population marked by a number of risk factors without offering definitive evidence of sources of risk.

The finding of greater self-reported fever among Zambians relative to other venue-goers is consistent with evidence from an earlier case–control study linking malaria to travel to Namibia’s northern border [18]. Greater cross-border interventions are needed, including targeted prevention and surveillance and improved coordination to ensure access to prevention information, and timely testing and treatment for individuals in Namibia who travel to Zambia and Angola.

The findings have other important limitations. Other types of venues, such as construction and agricultural work sites (identified in the formative research but not included in this study), could be associated with malaria risk and should be examined by future research. Due to time constraints, rates of venue attendance were not assessed by direct observation ahead of the study in order to inform the sampling plan; this may have contributed in part to failure to meet sample size targets [19]. There was considerable non-response on the household IRS measure, which was more common among younger respondents (*χ*^*2*^*P* = 0.002), suggesting that youth may not be able to provide information needed to characterize malaria risk at their residence. It was not possible to assess the degree of bias in prevalence estimates due to potentially greater participation by individuals who may have been motivated to obtain free malaria testing because they were ill; future venue-based studies should record reasons for participation and refusal.

TLS was developed in the context of HIV in urban settings dense with venues and venue-goers [6, 7], whereas transmission in malaria elimination settings tends to occur in rural, poor, low-population areas. This presents a challenge for reaching the planned sample size; future TLS surveillance should consider longer data collection periods and expanding the study area to compensate.

## Conclusion

In the global push to eliminate malaria, many countries have identified high-risk populations that play an important role in sustaining transmission yet are not easily reached by conventional surveillance and control strategies [5]. The present study suggests that in a region of continuing malaria transmission in Namibia, targeted prevention at night-time venues offers a strategy complementary to the largely household-based strategies in place, such as widespread provision of LLINs, IRS and strengthening diagnostic capacity at health services. Bars and evening churches provide efficient access to individuals who tend to be out at night and are characterized by several known risk factors. Importantly, they also provide more efficient access to young males (at bars) and overnight travelers (at bars and churches) than household surveys, and to individuals who also may be missed by passive surveillance, as they are less likely to present to clinics when ill relative to the general population.

Strategies that should be considered at these sites include providing information on malaria prevention and testing, providing on-site testing and treatment, and distributing LLINs. Additional research is needed to clarify which venues actually play a role in transmission, in order to prioritize venue-focused interventions such as IRS. Such research should include gathering vector data around suspected venues, conducting TLS during a greater portion of malaria season in order to increase power to detect elevated risk, and including questions about venue attendance in malaria indicator surveys.

While individuals who engage in outdoor social and occupational activities are increasingly seen as a target for onsite testing and treatment [3, 4, 20], improvements in the limited diagnostic value of RDTs are required to enhance the effectiveness and cost-effectiveness of such interventions.

## Data Availability

The dataset supporting the conclusions of this article can be requested from the Namibia Ministry of Health and Social Services.
